# A combination of stromal PD‐L1 and tumoral nuclear β‐catenin expression as an indicator of colorectal carcinoma progression and resistance to chemoradiotherapy in locally advanced rectal carcinoma

**DOI:** 10.1002/cjp2.285

**Published:** 2022-06-27

**Authors:** Hiroyuki Takahashi, Hirono Watanabe, Miki Hashimura, Toshihide Matsumoto, Ako Yokoi, Mayu Nakagawa, Yu Ishibashi, Takashi Ito, Kensuke Ohhigata, Makoto Saegusa

**Affiliations:** ^1^ Department of Pathology Kitasato University School of Medicine Sagamihara Japan; ^2^ Department of Pathology Kitasato University School of Allied Health Science Sagamihara Japan

**Keywords:** PD‐L1, PD‐1, β‐catenin, colorectal carcinoma, cancer stem cell, neoadjuvant chemoradiotherapy

## Abstract

Programmed cell death‐1 (PD‐1) and its ligand (PD‐L1) are significant mediators of immune suppression in the tumor microenvironment. We focused on the immunological impact of PD‐1/PD‐L1 signaling during tumor progression in colorectal carcinoma (CRC) and its association with resistance to neoadjuvant chemoradiotherapy (NCRT) in locally advanced rectal carcinoma (LAd‐RC). Histopathological and immunohistochemical analyses of 100 CRC cases (including 34 RC) without NCRT and 109 NCRT‐treated LAd‐RC cases were performed. Membranous tumoral PD‐L1 expression was identified in 9 of 100 (9%) CRC cases, including 1 of 34 (2.9%) RC cases, but PD‐L1 immunopositivity was not associated with any clinicopathological factors, with the exception of deficient mismatch repair (dMMR) status. In contrast, stromal PD‐L1+ immune cells, which frequently exhibited coexpression of PD‐1 and CD8 markers, were significantly correlated with tumor vessel invasion, nuclear β‐catenin+ tumor budding cancer stem cell (CSC)‐like features, and unfavorable prognosis. In the LAd‐RC cases, stromal CD8+ (but not PD‐L1+) immune cell infiltration in pretreatment‐biopsied samples was significantly and positively associated with therapeutic efficacy. After NCRT, tumoral PD‐L1 expression was observed in only 2 of 83 (2.4%) tumors, independent of dMMR status, whereas high stromal PD‐L1+ and tumoral nuclear β‐catenin positivity were significantly linked to a poor response to NCRT and high tumor budding features. In addition, high stromal PD‐L1 immunoreactivity was significantly associated with poorer overall survival. In conclusion, a combination of stromal PD‐L1+ immune cells and nuclear β‐catenin+ tumor budding may contribute to tumor progression in CRC and resistance to NCRT in LAd‐RC, through formation of niche‐like lesions that exhibit immune resistance and CSC properties.

## Introduction

Colorectal carcinoma (CRC) is one of the most common malignancies and a major contributor to carcinoma‐related deaths in Asia [1]. A strong lymphocytic reaction, including high‐density T‐cell subpopulation infiltration, is generally associated with favorable clinical outcome in CRC, supporting a major role for T‐cell‐mediated immunity in tumor suppression [2, 3]. Immune checkpoint mechanisms may also play an important role in suppressing the anti‐tumor T‐cell‐mediated immune response within the tumor microenvironment [4].

Programmed cell death protein 1 (PD‐1) is an inhibitory immune checkpoint molecule that is expressed on the surface of activated T‐cells to regulate proliferation and activation [5]. The dominant ligand for PD‐1, programmed death ligand 1 (PD‐L1), is expressed in activated T‐cells, B‐cells, dendritic cells, macrophages, endothelial cells, and certain tumor types [6]. Binding of PD‐L1 to PD‐1 can attenuate the cellular immune response by reducing apoptosis in regulatory T‐cells or by promoting T‐cell exhaustion [7, 8], and prominent clinical benefits for blockage of the PD‐1/PD‐L1 signaling have been demonstrated in several malignancies [9, 10]. However, the exact role of PD‐1/PD‐L1 signaling in CRC is less clear, with some published studies reporting conflicting results. For example, PD‐L1 expression is paradoxically associated with improved survival in mismatch repair (MMR)‐proficient CRC [11], whereas PD‐L1 can be used to identify CRC patients with high risk of metastasis and poor prognosis [12].

Neoadjuvant chemoradiotherapy (NCRT) for locally advanced rectal carcinoma (LAd‐RC) before total mesorectal excision is carried out to induce downstaging, downsizing, and significant changes in histological characteristics; this increases resectability and reduces postoperative locoregional recurrence rates [13, 14]. However, such cytotoxic therapies are associated with the development of a proinflammatory tumor microenvironment, leading to the activation of tumor‐specific adaptive immunity [15, 16]. Recently, strategies to combine NCRT with immune checkpoint inhibitors have been investigated as a means of improving the response rates to PD‐1/PD‐L1 antibody treatment [17, 18].

In this study, we set out to first examine the expression and significance of several immune cell‐related markers including PD‐L1 and PD‐1, as well as nuclear β‐catenin‐related tumor budding features, during tumor progression in CRC without NCRT. We also investigated whether there was an association between NCRT resistance and immunological impact in patients with LAd‐RC who had undergone NCRT.

## Materials and methods

### Clinical cases

We retrospectively evaluated pathological specimens from 100 consecutive CRC patients who had undergone surgery between 2011 and 2012, at Kitasato University Hospital, according to the criteria of the Japanese Classification of Colorectal, Appendiceal, and Anal Carcinoma and the TNM classification [19, 20]. In addition to the above CRC cases, we also selected 109 LAd‐RC patients, with tumors defined as clinical T3 or T4, N0 to N2, and M0, from among those patients who underwent NCRT (45 Gy given in 25 daily doses of 1.8 Gy as radiotherapy and administration of S‐1 and an irinotecan‐containing regimen as chemotherapy [21]) followed by total mesorectal excision at least 6 weeks later at Kitasato University Hospital during the period from 2006 to 2014. The pretreatment‐biopsied samples from LAd‐RC patients receiving NCRT were also investigated. In resected tumor samples, one block section including tumor progression showing the parts with the deepest invasion in each case was selected for the evaluation of histopathological and immunohistochemical findings.

Tumor vascular invasion was also evaluated according to the criteria of the Japanese Classification of Colorectal, Appendiceal, and Anal Carcinoma [19] using Elastic van Gieson staining. In addition, regional lymph nodal and distant metastases were evaluated by histopathological and radiographical examinations. All tissues were routinely fixed in 10% formalin and processed for embedding in paraffin wax. This study was approved by the Kitasato University Medical Ethics Committee (B19‐155).

### Evaluation of histopathological findings

To evaluate the heterogenous distribution of infiltrating immune cells in tumor tissues including the surrounding stroma in CRC without NCRT, they were subcategorized into three groups on the basis of the tumor sizes as follows: inner, upper one‐third of the lesion area; middle, middle one‐third; and outer, lower one‐third (supplementary material, Figure S1). LAd‐RC cases receiving NCRT were also subclassified into three groups on the basis of the therapeutic efficacy (TE) of NCRT, as follows: TE: grade (G) 1, necrosis or degeneration of tumor cells present in less than two‐thirds of the entire lesion; TE: G2, necrosis/degeneration present in more than two‐thirds of the lesions; and TE: G3, no tumor elements, according to the criteria of the Japanese Classification of Colorectal, Appendiceal, and Anal Carcinoma [19]. Sixty‐one cases were subclassified as TE: G1, 28 as TE: G2, and 20 as TE: G3.

Tumor budding (BD) was defined as the presence of individual and/or small groups of tumor cells at the invasive fronts as described previously [22]. The BD score was evaluated according to the criteria of the Japanese Classification of Colorectal, Appendiceal, and Anal Carcinoma [19]. In brief, we scanned all areas of the slides to observe the distribution of BD features in tumor lesions. Within the field of view, an area containing predominantly BD (hot‐spot) was selected and the buds were counted using a ×20 objective. The BD features were then subcategorized into three groups as follows: BD: 1, less than four individual and/or small groups of tumor cells at the invasive fronts; BD: 2, five to nine; and BD: 3, more than 10. Among 109 cases receiving NCRT, 20 with TE: G3 had no tumor elements due to TE, whereas one with TE: G2 could not evaluate the residual tumors due to loss of elements in hematoxylin and eosin‐stained specimen. Finally, 58 cases were subclassified as BD: 1, 23 as BD: 2, and 19 as BD: 3 in CRC without NCRT, whereas 41 cases were scored as BD: 1, 15 as BD: 2, and 32 as BD: 3 in LAd‐RC patients receiving NCRT.

### Immunohistochemistry

Immunohistochemistry (IHC) was performed using a combination of microwave oven heating and immuno‐enzyme polymer (Nichirei Bioscience Inc, Tokyo, Japan) methods. Antibodies used in this study are shown in supplementary material, Table S1. The immunoreactions were visualized using 3,3′‐diaminobenzidine or cobalt chloride.

PD‐L1 expression was evaluated according to the criteria of Rosenbaum *et al* [23]. In brief, tumor cells were considered PD‐L1‐positive if more than 5% of them had membranous (but not cytoplasmic) staining of any intensity. For quantification of immune cells that infiltrated the stroma of CRC without NCRT, labeling indices (LIs) of PD‐L1‐, PD‐1‐, CD4‐, CD8‐, and CD68‐immunopositive immune cells were calculated by counting at least 500 stromal cells. The LIs were then expressed as percentages. For intratumoral immunopositive immune cell infiltrations, five fields within each intraepithelial lesion were randomly selected. The number of immunopositive immune cells was also then calculated by counting the mean number per high‐power field. We also scored the above immune markers and nuclear β‐catenin immunoreactivity in LAd‐RC cases receiving NCRT, and quantified nuclear β‐catenin immunoreactivity in tumor invasive fronts in cases of CRC without NCRT. In brief, the percentage of immunopositive cells in the total stromal or tumor cell population was subdivided into five categories as follows: 0, all negative; 1, <30% of positive cells; 2, 30–50%; 3, 50–70%; and 4, >70% of positive cells. The immunointensity was also subclassified into four groups, as follows: 0, negative; 1+, weak; 2+, moderate; and 3+, strong. IHC scores were produced by multiplication of the two values. For MMR status, cases with complete loss or subclonal patterns of staining were defined as MMR‐deficient as described by Watkins *et al* [24]. The evaluation of IHC findings was conducted by three observers (HT, HW, and MH).

### Immunofluorescence

To examine the coexpression of several immune cell‐related markers, sections were incubated with primary antibodies, and fluorescein isothiocyanate‐ or rhodamine‐labeled anti‐mouse or rabbit IgGs (Molecular Probes, Eugene, OR, USA) were used as secondary antibodies. Details of the antibodies are shown in supplementary material, Table S1.

### Statistical analysis

Comparative data were analyzed using the Mann–Whitney *U*‐test, chi‐square test, or Spearman's rank correlation coefficient, as appropriate. To evaluate the prognostic significance of immune cell‐related factors, the LIs were divided into two categories (high and low) based on the cut‐off values (mean − standard deviation) (Tables 1 and 2). Overall survival (OS) was calculated as the time between onset and death or the date of the last follow‐up evaluation. Progression‐free survival (PFS) was also examined from the onset of treatment until relapse, disease progression, or last follow‐up evaluation. OS and PFS were estimated using the Kaplan–Meier method, and statistical comparisons were made using the log‐rank test. Univariate and multivariate analyses were also performed using the Cox proportional hazards regression model. The cutoff for statistical significance was set as *p* < 0.05.

**Table 1:** Correlation between PD‐L1 expression and clinicopathological factors in carcinomatous and the outer stroma of CRC without NCRT

		PD‐L1 (carcinoma)				PD‐L1 (outer stroma)		
		High (≧5%)	Low (<5%)			High (≧30%)	Low (<30%)	
	*n*	*n* (%)	*n* (%)	*P* value	*n*	*n* (%)	*n* (%)	*P* value
Age (years)								
≧68	48	5 (5.5)	43 (47.3)	0.9	52	46 (48.4)	6 (6.3)	0.8
<68	43	4 (4.4)	39 (42.9)		43	32 (33.7)	11 (11.6)	
Gender								
Male	55	5 (5.5)	50 (54.9)	0.8	56	26 (27.6)	30 (31.9)	0.7
Female	36	4 (4.4)	32 (35.2)		38	19 (20.2)	19 (20.2)	
Location								
Left side	38	7 (7.7)	31 (34.1)	0.02	38	16 (16.8)	22 (23.2)	0.3
Right side	53	2 (2.2)	51 (56.0)		57	30 (31.6)	27 (28.4)	
Histology								
Well/mod	76	9 (11.1)	67 (82.7)	0.4	79	38 (44.7)	41 (48.2)	0.9
Muc/pap	5	0 (0)	5 (6.2)		6	3 (3.5)	3 (3.5)	
Clinical stage								
I	7	1 (1.9)	6 (11.3)	0.6	15	6 (6.4)	9 (9.6)	0.6
II	20	2 (3.8)	18 (34.0)		37	16 (17)	21 (22.3)	
IIIa	14	0 (0)	14 (26.4)		25	13 (13.8)	12 (12.7)	
IIIb	12	1 (1.9)	11 (20.8)		17	10 (10.6)	7 (7.4)	
LN meta								
Positive	40	2 (2.2)	38 (41.8)	0.2	42	23 (24.5)	19 (20.2)	0.2
Negative	51	7 (7.7)	44 (48.4)		52	22 (23.4)	30 (31.9)	
Ly infiltration								
Positive	64	6 (6.6)	58 (63.7)	0.8	68	39 (41.4)	29 (30.9)	0.003
Negative	27	3 (3.3)	24 (26.4)		26	6 (6.4)	20 (21.2)	
V infiltration								
Positive	77	7 (7.7)	70 (76.9)	0.5	79	42 (44.7)	37 (39.4)	0.02
Negative	14	2 (2.2)	12 (13.2)		15	3 (3.2)	12 (12.8)	

Cut‐off values for each category were defined as mean − standard deviation values.

LN meta, lymph node metastasis; Ly, lymph vessel; *n*, number of cases; muc/pap, mucinous/papillary; V, venous vessel; well/mod, well/moderately differentiated.

**Table 2:** Univariate and multivariate analyses for OS and PFS in CRC without NCRT

Univariate analysis					Multivariate analysis				
Variables	Cutoff	Log‐rank c^2^	*P* value	Unfavorable factor	Variables	Cutoff	Hazard ratio	95% CI	*P* value
OS					OS				
PD‐L1 (carcinoma)	≧5/<5	0.005	0.9		PD‐1 (outer stroma)	0.51	0.3	0.1–0.9	0.02
PD‐L1 (outer stroma)	10.3	0.07	0.8		CD8 (outer stroma)	26.2	0.2	0.08–0.8	0.01
PD‐1 (outer stroma)	0.5	9.6	0.002	Low score	Age	68/69	1	1–1.1	0.05
CD4 (outer stroma)	18.3	0.3	0.6		Clinical stage	I・II/IIIa・IIIb	0.3	0.1–0.9	0.03
CD8 (outer stroma)	26.2	5.2	0.02	Low score	LN metastasis	−/+	0.3	0.1–0.9	0.03
CD68 (outer stroma)	14	0.0	0.9		Ly involvement	−/+	0.1	0.02–0.9	0.04
MMR status	prof/def	ND	ND		BD score	BD: 1/BD: 2, 3	0.4	0.1–1.5	0.18
Age	68/69	5.1	0.02	Older					
Gender	M/F	0.0	0.8						
Location	Right/left	0.6	0.5						
Histology	pap・muc/tub	0.9	0.4						
Clinical stage	I・II/IIIa・IIIb	7.2	0.007	IIIa・IIIb					
LN metastasis	−/+	7.2	0.007	+					
Ly involvement	−/+	5.5	0.02	+					
Venous involvement	−/+	NE	NE						
BD score	BD: 1/BD: 2, 3	10.8	0.01	BD: 2, 3					
PFS					PFS				
PD‐L1 (carcinoma)	≧5/<5	1.36	0.24		PD‐1 (outer stroma)	0.51	0.4	0.2–0.9	0.03
PD‐L1 (outer stroma)	10.3	0.6	0.4		Clinical stage	I II/IIIa IIIb	0.5	0.3–0.8	0.004
PD‐1 (outer stroma)	0.51	10.7	0.001	Low score	LN metastasis	−/+	0.9	0.3–0.8	0.004
CD4 (outer stroma)	18.365	0.02	0.9		Ly involvements	−/+	0.5	0.5–1.6	0.8
CD8 (outer stroma)	26.2	2.7	0.1		Venous involvement	−/+	1	0.5–1.8	0.9
CD68 (outer stroma)	14.002	1.3	0.2		BD score	BD: 1/BD: 2, 3	0.5	0.1–1.7	0.2
MMR status	prof/def	1.04	0.3						
Age	68/69	2.1	0.1						
Gender	M/F	1.1	0.3						
Location	Right/left	0.2	0.7						
Histology	pap・muc/tub	0.5	0.5						
Clinical stage	I・II/IIIa・IIIb	16.2	<0.0001	IIIa・IIIb					
LN metastasis	−/+	16.2	<0.0001	+					
Ly involvement	−/+	9	0.003	+					
Venous involvement	−/+	3.7	0.05	+					
BD score	BD: 1/BD: 2, 3	6.5	0.01	BD: 2, 3					

Cut‐off values for each category were defined as mean − standard deviation values. Clinical stage refers to the criteria of the Japanese Classification of Colorectal, Appendiceal, and Anal Carcinoma.

LN, lymph node; Ly, lymph vessel; ND, not detected; NE, not evaluated; pap・muc, papillary・mucinous; prof/def, proficient/deficient.

## Results

### PD‐L1 is predominantly expressed in stromal immune cells in CRC without NCRT

**Figure 1:**
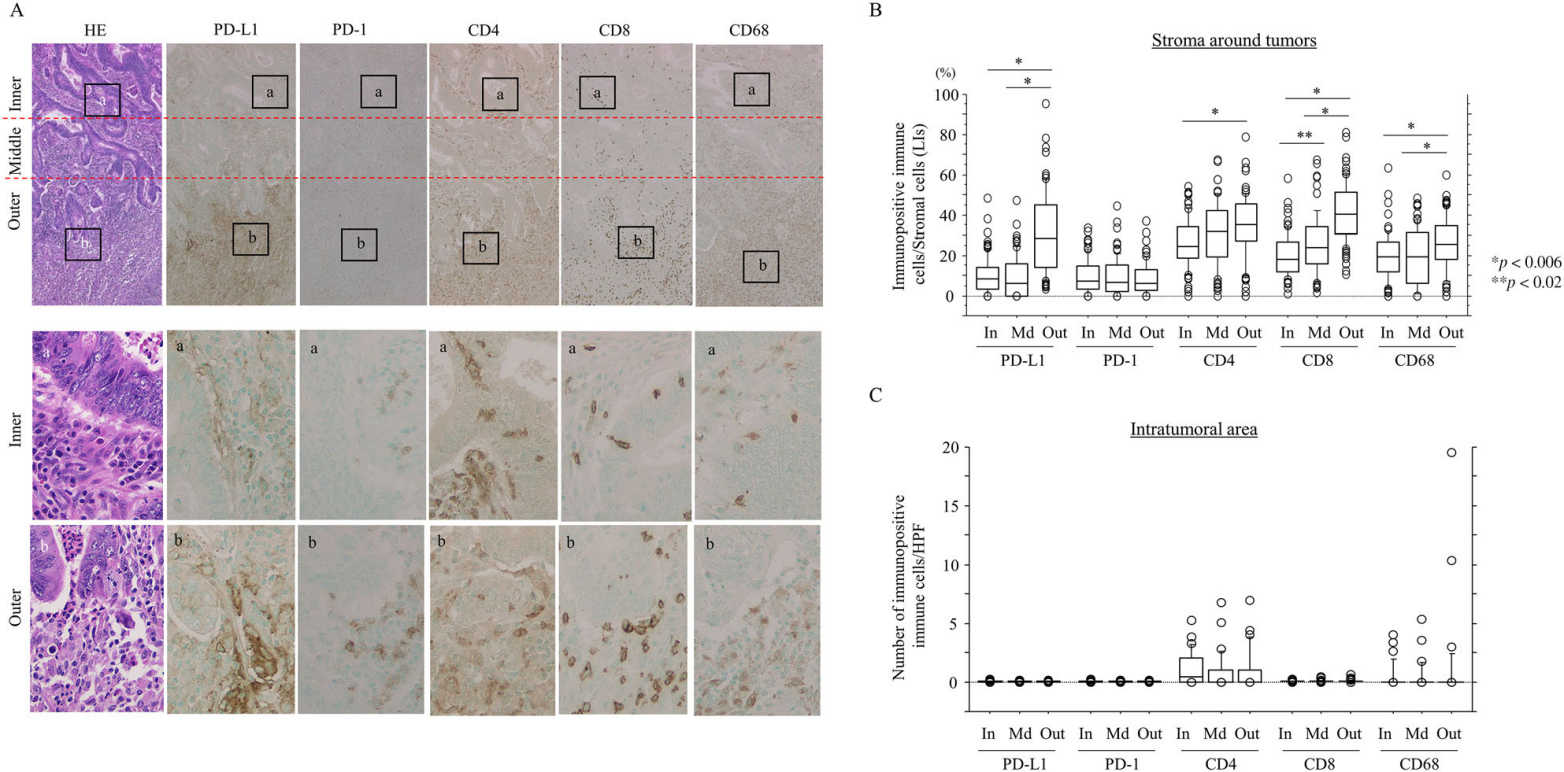
High density of infiltrating immune cells in the outer stroma of CRC without NCRT. (A) Staining with hematoxylin and eosin (HE) or IHC for the indicated immune cell‐related markers in CRC without NCRT. Note the high density of infiltrating immune cells expressing the indicated markers in the outer stroma compared to the inner and middle areas. Closed boxes in the upper panels are magnified in the lower panels. Original magnification, ×100 (upper) and ×400 (lower). (B) LIs of immunopositive immune cells for the indicated markers in the inner (In), middle (Md), and outer (Out) stroma in CRC samples. The data shown are means ± SDs. (C) Number of immune cells immunopositive for the indicated markers per high‐power field (HPF) in the In, Md, and Out intratumoral lesions in CRC samples. The data shown are means ± SDs.

Representative IHC images for PD‐L1, PD‐1, CD4, CD8, and CD68 in CRC without NCRT are illustrated in Figure 1A. No significant differences were observed in the expression of any of the IHC markers investigated in infiltrating immune cells or tumor cells between patients with colon carcinoma and RC (supplementary material, Figure S2). In addition, there were no correlations between CD3, CD4, or CD8 immunoreactivities in the outer stroma of CRC (supplementary material, Figure S3).

With the exception of PD‐1, all the markers showed significant region‐specific differences in the LIs. Notably, the LIs for PD‐L1 were significantly higher in the outer stroma than in the inner and middle stroma (Figure 1B), along with a significantly higher ratio of PD‐L1 immunoreactivity relative to PD‐1 and CD4 values in the outer stroma (supplementary material, Figure S4). In contrast, such differences were not observed in intratumoral infiltrating immune cells (Figure 1C). Frequent coexpression of these immune cell‐related markers was also observed in infiltrating immune cells in the outer stroma (supplementary material, Figure S5A), with a positive correlation between PD‐L1+ and CD8+, but not PD‐1, CD4, and CD68, immune cells (supplementary material, Figure S5B).

Membranous PD‐L1 expression in tumor cells was also identified in 9 of 100 (9.0%) CRC cases, including 1 of 34 (2.9%) RC cases (supplementary material, Figure S5). As CRC patients with deficient MMR (dMMR) generally respond better to anti‐PD‐1/PD‐L1 immunotherapy than their MMR‐proficient counterparts [25, 26], we investigated whether there was an association between dMMR status and PD‐L1 expression in CRC. Using IHC, we classified 10 of 100 (10%) tumors as having dMMR; these cases were associated with high PD‐L1 expression specifically in the carcinoma cells, but such association was not observed in stromal PD‐L1 status (supplementary material, Figure S6 and Table S2).

### Stromal PD‐L1+ immune cell infiltrations are positively correlated with tumor budding and tumor vascular invasion in CRC without NCRT

**Figure 2:**
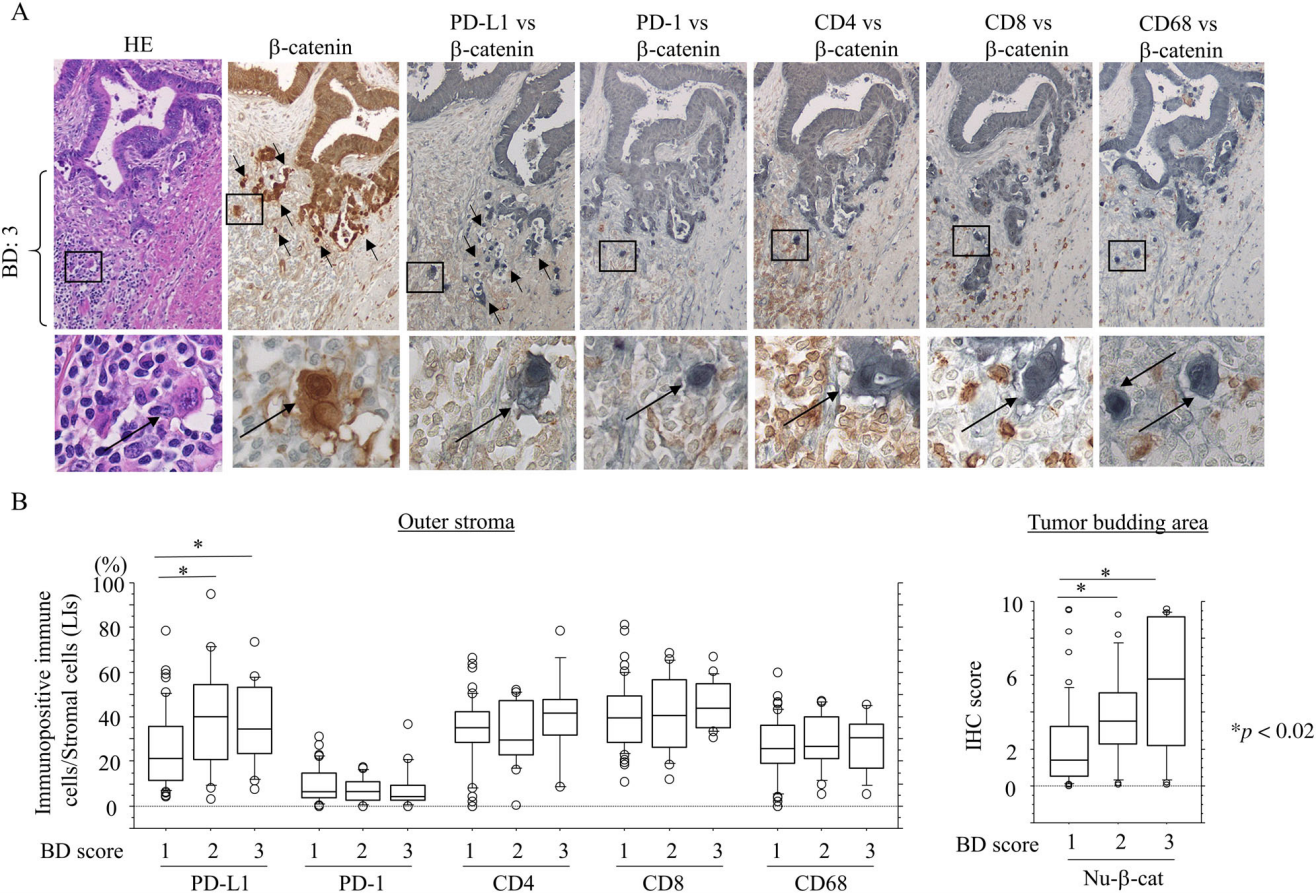
Relationship between stromal immune cell infiltrations and nuclear β‐catenin‐positive tumor buds in CRC without NCRT. (A) Staining with hematoxylin and eosin (HE), IHC for β‐catenin (visualized using 3,3′‐diaminobenzidine), and double IHC for β‐catenin (visualized using sodium cobalt) and the indicated immune cell‐related markers (visualized using 3,3′ ‐diaminobenzidine) in CRC samples with a tumor budding score (BD): 3 without NCRT. Note the infiltrating immune cells expressing the indicated markers around the nuclear β‐catenin+ tumor budding cells (indicated by arrows). Closed boxes in the upper panels are magnified in the lower panels. Original magnification, ×100 (upper) and ×400 (lower). (B) Association between LIs of immunopositive immune cells expressing the indicated markers (left) or IHC score of nuclear β‐catenin (Nu‐β‐cat) (right) and BD score in the outer stroma of CRC samples. The data shown are means ± SDs.

As nuclear β‐catenin accumulation contributes to tumor budding in CRC [27, 28], we examined whether there were any associations between stromal immune cell infiltrations, nuclear β‐catenin+ tumor cells, and tumor budding scores in our cohort. Representative images of immune cell‐related molecules and β‐catenin in CRC cases (with high tumor budding features) without NCRT are shown in Figure 2A. In the outer stroma, average stromal PD‐L1 LIs and nuclear β‐catenin scores were significantly higher in tumors with BD: 2 and BD: 3 compared to those with BD: 1 status (Figure 2B). In addition, stromal (but not carcinomatous) PD‐L1 LIs in the outer (but not inner or middle) stroma were significantly associated with tumor lymph and venous invasions, whereas there was no correlation between clinicopathological factors and the status of PD‐1, CD4, CD8, or CD68 (Table 1 and supplementary material, Table S3).

### Stromal PD‐1 and CD8, but not PD‐L1, are independent prognostic factors in CRC without NCRT

**Figure 3:**
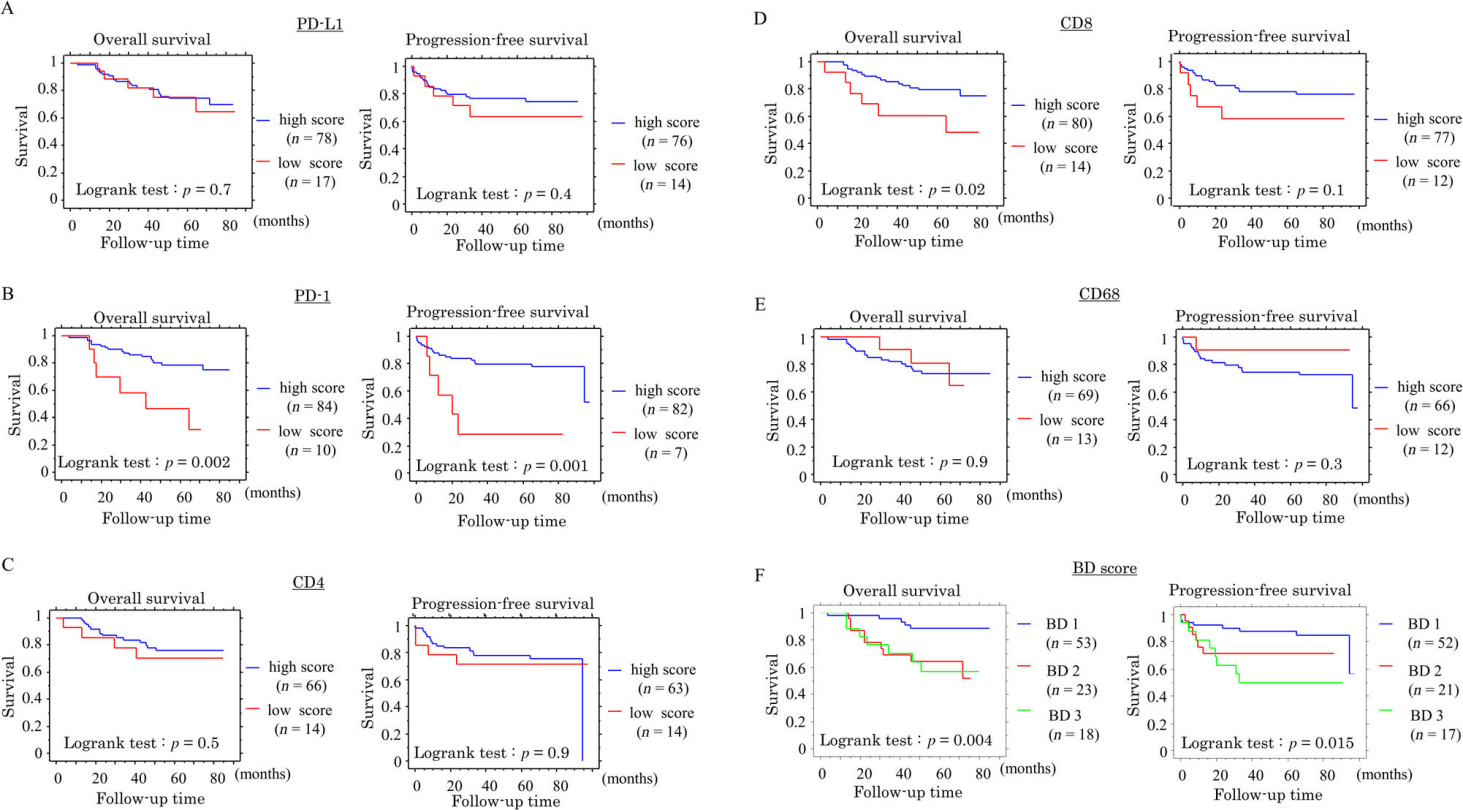
Relationship between immune cell‐related markers, BD score, and prognosis in CRC without NCRT. OS (left) and PFS (right) relative to PD‐L1 (A), PD‐1 (B), CD4 (C), CD8 (D), CD68, and BD score (F) in CRC without NCRT. *n*, number of cases.

In CRC without NCRT patients, Kaplan–Meier analysis showed that high stromal PD‐1 and high CD8 values in the outer stroma were associated with a more favorable OS and/or PFS compared to patients with low PD‐1 and low CD8 values. OS and PFS were also superior in patients with a BD: 1 score compared to those with BD: 2 or BD: 3 scores. There was no association between OS or PFS and stromal PD‐L1+ immune cells (Figure 3). Univariate Cox regression revealed that stromal PD‐1+ and CD8+ immune cell infiltrations in the outer stroma, as well as age, clinical stage, lymph node metastasis, tumor lymph or venous invasions, and BD score, were significant prognostic factors for OS or PFS. Multivariate analysis also showed that stromal PD‐1, CD8, clinical stage, lymph node metastasis, and tumor lymph invasions were significant and independent prognostic factors for OS or PFS (Table 2).

### Stromal PD‐L1 and tumoral nuclear β‐catenin expression are associated with TE and tumor budding in LAd‐RC patients receiving NCRT

Representative IHC findings for PD‐L1, PD1, CD4, CD8, and CD68 in pretreatment‐biopsied samples from LAd‐RC patients before NCRT are shown in supplementary material, Figure S7A. Only CD8 LIs in stroma around tumors were significantly higher in patients who responded well to NCRT (TE: G3) compared to those patients with a G1 or G2 TE. In contrast, there was no association between any of the intramural immune cells in pretreatment‐biopsied RC samples and TE grades in the corresponding resected tumors (supplementary material, Figure S7B).

**Figure 4:**
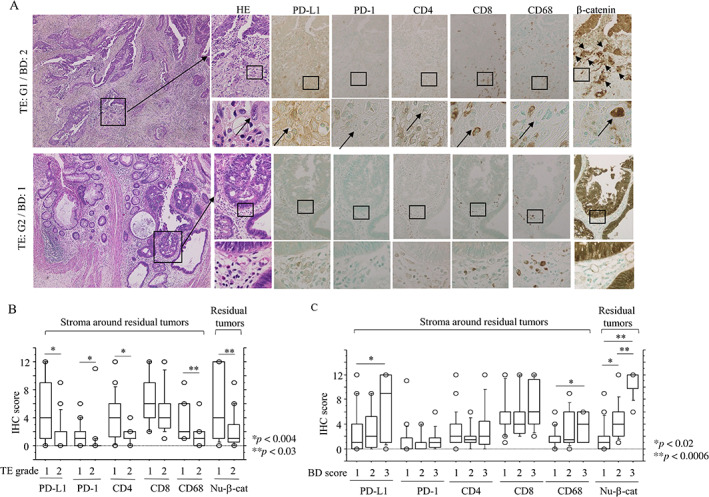
Expression of immune cell‐related molecules in resected LAd‐RC after NCRT. (A) Staining with hematoxylin and eosin (HE) and IHC for the indicated immune cell‐related markers and tumoral β‐catenin in samples of LAd‐RC cases that responded poorly to NCRT and had moderate budding features (TE: G1/BD: 2) (upper panels) or responded well and had low budding scores (TE: G2/BD: 1) (lower panels). Note the high density of infiltrating immune cells expressing the indicated markers around the tumor budding cells (indicated by arrows) in the stroma of RC with TE: G1/BD: 2 (upper panels). For two RC cases, the closed boxes in the left panels are magnified in the right panels. Closed boxes in the upper panels are also magnified in the lower panels in each LAd‐RC case. Original magnification, ×40 (left), ×100 (upper), and ×400 (lower). (B, C) Relationship between IHC score for immune cells expressing the indicated molecules, tumoral nuclear β‐catenin (Nu‐β‐cat), TE grade (B), and BD score (C) in samples from LAd‐RC patients treated with NCRT. The data shown are means ± SDs.

**Figure 5:**
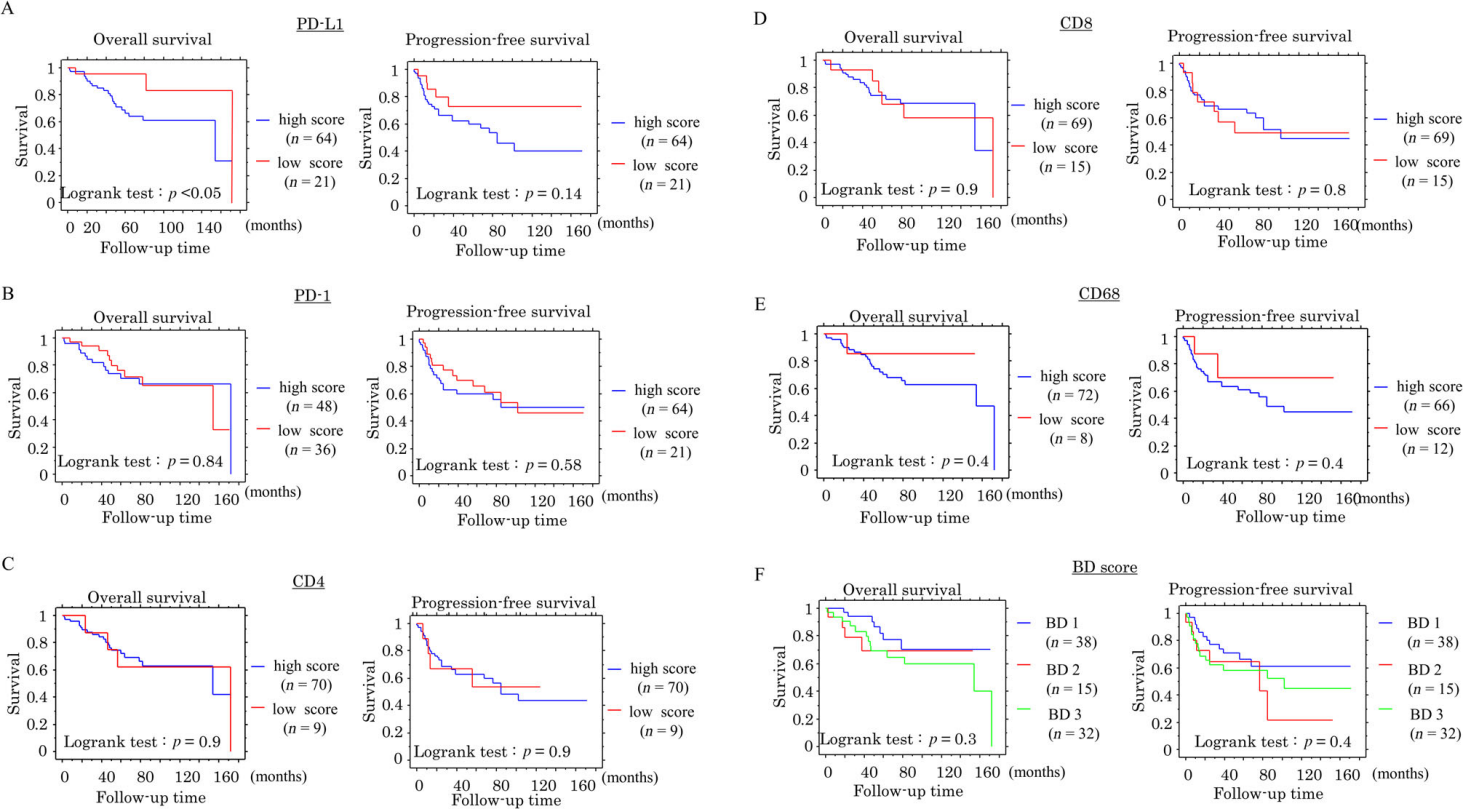
Relationship between immune cell‐related markers, BD score, and prognosis in LAd‐RC patients who received NCRT. OS (left) and PFS (right) relative to PD‐L1 (A), PD‐1 (B), CD4 (C), CD8 (D), CD68, and BD score (F) in LAd‐RC after NCRT. *n*, number of cases.

The results of IHC for stromal immune cells and tumoral β‐catenin in resected LAd‐RCs after NCRT are shown in Figure 4A. There was an inverse correlation between TE grade and BD score (supplementary material, Table S4). Tumoral PD‐L1 expression was observed in 2 of 83 (2.4%) LAd‐RC cases, where one was subcategorized as TE: G1/dMMR and the remaining case was classified as TE: G2/proficient MMR. Average IHC scores for most stromal immune cell‐related markers and tumoral nuclear β‐catenin were significantly lower in patients who responded well to NCRT (TE: G2) compared to those who responded poorly (TE: G1; Figure 4B). The IHC scores of stromal PD‐L1+ and CD68+ immune cells and tumoral nuclear β‐catenin after NCRT were also significantly higher in LAd‐RC cases with a high BD score (BD: 3) compared to tumors with low or moderate scores (BD: 1 or BD: 2) (Figure 4C). Kaplan–Meier analysis showed that patients with high stromal PD‐L1 LIs had unfavorable OS compared to patients with low stromal PD‐L1 values (Figure 5). This association was also identified following univariate Cox regression analysis, although the difference did not reach the required level of significance (supplementary material, Table S5).

## Discussion

Here, we provide clear evidence that the proportion of infiltrating immune cells expressing PD‐L1, CD4, CD8, or CD68 is significantly higher in the outer stroma (the distal third of the specimens) of CRC without NCRT compared to the inner and middle stroma; no such association was observed with regard to PD‐1+ immune cells. This may be due to the immunological reaction that is initiated once the tumor begins to invade the stroma through the tumor budding process. In addition, there were significantly more PD‐L1+ immune cells that coexpressed either PD‐1 or CD8 in the outer stroma. In general, PD‐L1 expression is a critical determinant of stromal cell immunomodulatory capacity, and the interaction between PD‐L1 and PD‐1 causes adaptive immune resistance through the inhibition of CD8+ T‐cell activation and proinflammatory cytokine secretion [29, 30]. As PD‐1 is a marker of effector T‐cells [31], it appears that the anti‐tumor immune response may be blunted in the outer stroma adjacent to tumor tissues due to the interaction with PD‐L1 and PD‐1 in CD8+ immune cells.

**Figure 6:**
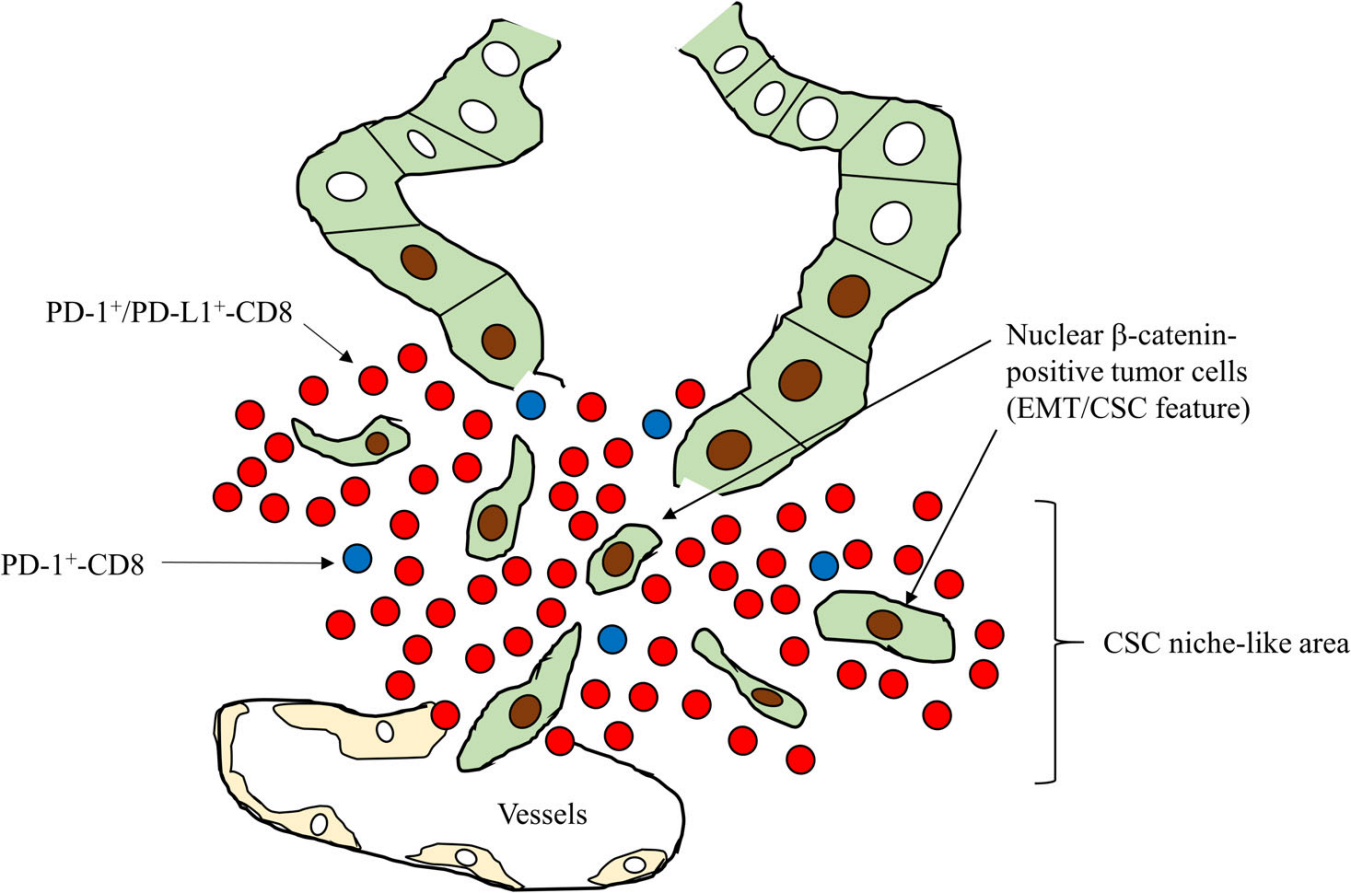
Schematic representation of the CSC niche‐like lesions in CRC patients and in LAd‐RC patients receiving NCRT. Densely infiltrating immune cells expressing PD‐L1, PD‐1, or CD8 and nuclear β‐catenin+ tumor budding cells are present in the outer stroma adjacent to tumor invasive fronts. Note the nuclear β‐catenin+ tumor cells, along with migration of PD‐L1+/PD‐1+/CD8+ immune cells, into vessels.

Interestingly, WNT/β‐catenin signaling affects cancer immunosurveillance across carcinoma types [32]. For example, tumor‐induced β‐catenin signaling inhibits the dendritic cell‐dependent cross‐sensitization of anti‐tumor cytotoxic T‐cells and also shifts infiltrating immune effector cells into an ‘immune‐tolerant’ state [33]. Moreover, nuclear β‐catenin signaling plays an important role in the induction of epithelial–mesenchymal transition (EMT)/cancer stem cell (CSC) properties through increased Slug and decreased E‐cadherin expression in endometrial and colon carcinoma cells [34, 35]. Leucine‐rich repeat‐containing G‐protein‐coupled receptor 5 (Lgr5), a target of Wnt pathway signaling and canonical marker of colorectal CSC, also contributes to the formation of budding features in CRC [36]. In our current study, stromal PD‐L1+ immune cell infiltration was significantly associated with nuclear β‐catenin+ tumor budding and tumor lymph/vascular invasion in CRC without NCRT. Given the evidence that EMT promotes stem cell properties and also generates cells with CSC‐like features [37], we suggest that a combination of PD‐L1+ immune cells and budding nuclear β‐catenin+ tumor cells may constitute CSC niche‐like lesions within CRC tissues. Consequently, sustained immune resistance and CSC properties will lead to tumor vascular invasion and disease progression (Figure 6).

In our results, high CD8+ (and PD‐1+) immune cell infiltrations in the outer stroma were significant and independent favorable prognostic factors for OS and PFS in CRC patients who did not receive NCRT. Moreover, high CD8+ immune cell infiltration in LAd‐RC biopsies before NCRT was positively correlated with TE after NCRT. This is consistent with the finding that CD8+ T‐cell density is strongly associated with positive clinical outcome in CRC patients [38]. In contrast, we could not demonstrate any prognostic significance of stromal PD‐L1 immunoreactivity in CRC without NCRT, despite the positive correlation between stromal PD‐L1+ and CD8+ expression. This discrepancy may be explained by the complex negative feedback mechanisms that govern PD‐L1 and CD8 expression. Specifically, proinflammatory cytokines produced by infiltrating T‐cells further increase PD‐L1 expression, which enhances immunosuppression in the tumor microenvironment [31].

In CRC without NCRT, tumoral PD‐L1 positivity is significantly associated with large tumor size, lymph node metastasis, high TNM stage, shorter disease‐free survival, and worse recurrence‐free survival, as well as MMR status [23, 38, 39, 40]. Furthermore, the expression of PD‐L1 is significantly increased in the tumors of RC patients who have received NCRT [16, 41], and a low PD‐L1 tumor proportion score was associated with inferior survival in RC patients undergoing NCRT [42]. However, our results indicate that, while tumoral PD‐L1 expression did correlate with MMR status, it was not associated with any of the clinicopathological factors, prognosis, or NCRT status in CRC or RC. The discrepancies between our results and those from the above studies are probably due to differences in study population (e.g. predominantly White, Black, or Asian subjects), design, or methods, including which anti‐PD‐L1 antibody and immunostaining technique were used. Further studies with a large number of patients will be required to clarify the significance of tumoral PD‐L1 expression.

More importantly, high stromal PD‐L1+ immune cell infiltration and high tumoral nuclear β‐catenin scores were significantly associated with a poor response to NCRT and high tumor budding features in LAd‐RC cases treated with NCRT. Moreover, we found that high stromal PD‐L1 immunoreactivity was significantly associated with poorer OS after NCRT, and that the TE of NCRT was inversely correlated with tumor budding scores. We therefore suggest that the CSC niche‐like lesions (characterized by high stromal PD‐L1+ immune cell infiltration and nuclear β‐catenin+ budding cells) may also serve as an indicator of resistance to NCRT in LAd‐RC patients (Figure 6).

In conclusion, the combination of stromal PD‐L1+ immune cells and nuclear β‐catenin+ tumor budding may have an important role in tumor progression in CRC and could contribute to the NCRT resistance in LAd‐RC. By extension, we suggest that combined therapy with NCRT and an immune checkpoint inhibitor will be effective in RC patients.

## Author contributions statement

HT, HW, MH and MS carried out the majority of the experiments, analyzed the data, and wrote the manuscript. They were helped by TM, AY, MN, YI and TI. All authors reviewed and approved the final manuscript.

## Supporting information


**Figure S1.** Subclassifications of inner, middle, and outer parts of a tumor lesion on the basis of tumor size
**Figure S2.** Differences in the expression of immune cell‐related markers between colon and rectal carcinomas
**Figure S3.** Relationship between CD3, CD4, and CD8 in CRC without NCRT
**Figure S4.** Ratios of PD‐L1 LIs relative to PD‐1, CD4, CD8, or CD68 LIs
**Figure S5.** Coexpression of immune cell‐related markers in infiltrating stromal immune cells in CRC without NCRT
**Figure S6.** Tumoral PD‐L1 and MMR expression in CRC without NCRT
**Figure S7.** Expression of immune cell‐related molecules in biopsied samples of LAd‐RC before NCRT
**Table S1.** Summary of antibodies used for IHC
**Table S2.** Association between carcinomatous and stromal PD‐L1 and MMR status in CRC without NCRT
**Table S3.** Correlation between IHC marker expression and clinicopathological factors in the outer stroma in CRC without NCRT
**Table S4.** Correlation between TE grade and BD score in LAd‐RC after NCRT
**Table S5.** Univariate analysis for OS and PFS in LAd‐RC after NCRT

## Data Availability

The datasets generated and/or analyzed during the current study are available from the corresponding author upon reasonable request.
