# Supplementary material for: A combination of stromal PD‐L1 and tumoral nuclear β‐catenin expression as an indicator of colorectal carcinoma progression and resistance to chemoradiotherapy in locally advanced rectal carcinoma
Source: J Pathol Clin Res. 2022 Jun 27;8(5):458–69. doi: 10.1002/cjp2.285 (PMC9353658; doi:10.1002/cjp2.285)
Supplement: Supplementary file 1 — Figure S1. Subclassifications of inner, middle, and outer parts of a tumor lesion on the basis of tumor size Figure S2. Differences in the expression of immune cell‐related markers between colon and rectal carcinomas Figure S3. Relationship between CD3, CD4, and CD8 in CRC without NCRT Figure S4. Ratios of PD‐L1 LIs relative to PD‐1, CD4, CD8, or CD68 LIs Figure S5. Coexpression of immune cell‐related markers in infiltrating stromal immune cells in CRC without NCRT Figure S6. Tumoral PD‐L1 and MMR expression in CRC without NCRT Figure S7. Expression of immune cell‐related molecules in biopsied samples of LAd‐RC before NCRT Table S1. Summary of antibodies used for IHC Table S2. Association between carcinomatous and stromal PD‐L1 and MMR status in CRC without NCRT Table S3. Correlation between IHC marker expression and clinicopathological factors in the outer stroma in CRC without NCRT Table S4. Correlation between TE grade and BD score in LAd‐RC after NCRT Table S5. Univariate analysis for OS and PFS in LAd‐RC after NCRT [file CJP2-8-458-s001.pdf]

A combination of stromal PD-L1 and tumoral nuclear β-catenin expression as an indicator of colorectal carcinoma progression and resistance to chemoradiotherapy in locally advanced rectal carcinoma

H Takahashi, H Watanabe *et al*. *J Pathol Clin Res* DOI: 10.1002/cjp2.285

Supplementary Material

Supplementary Figures S1-S7
Supplementary Tables S1-S5

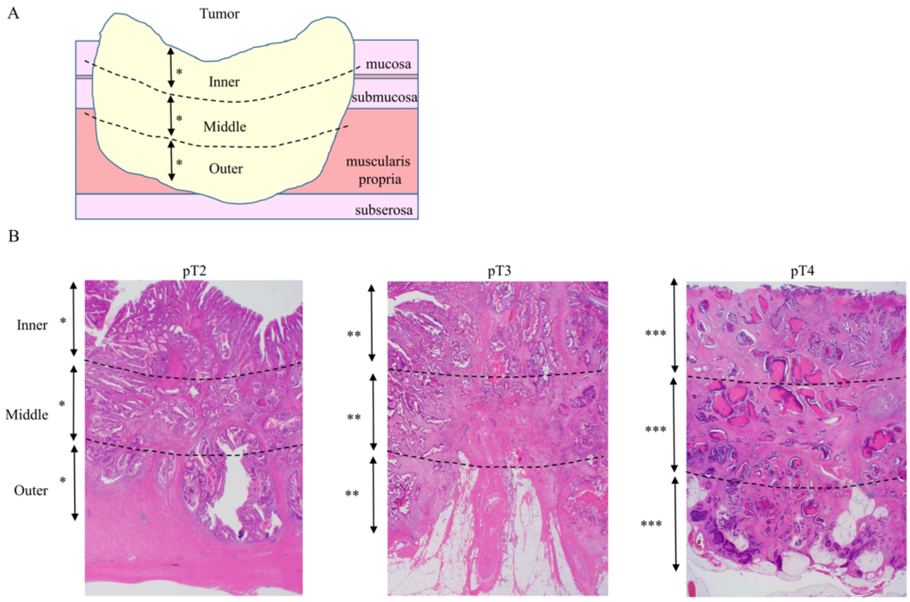

Figure S1. (A) A diagram and (B) pT2 (left), pT3 (middle), and pT4 (right) CRCs showing the three subclassifications of inner, middle, and outer parts of a tumor lesion on the basis of tumor size. *, **, ***, one-third of the lesions.

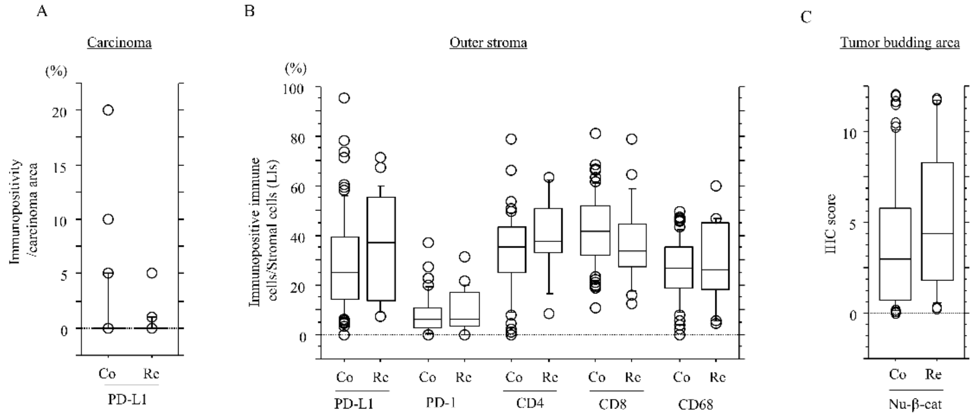

Figure S2. **Differences in the expression of immune cell-related markers between colon and rectal carcinomas.** (A) PD-L1 immunopositivity in tumor cells, (B) labeling indices (LIs) of immunopositive immune cells for the indicated markers in the outer stroma, and (C) nuclear β-catenin score in the tumor budding area in colon (Co) and rectal carcinomas (Re) without NCRT. The data shown are means ± SDs.

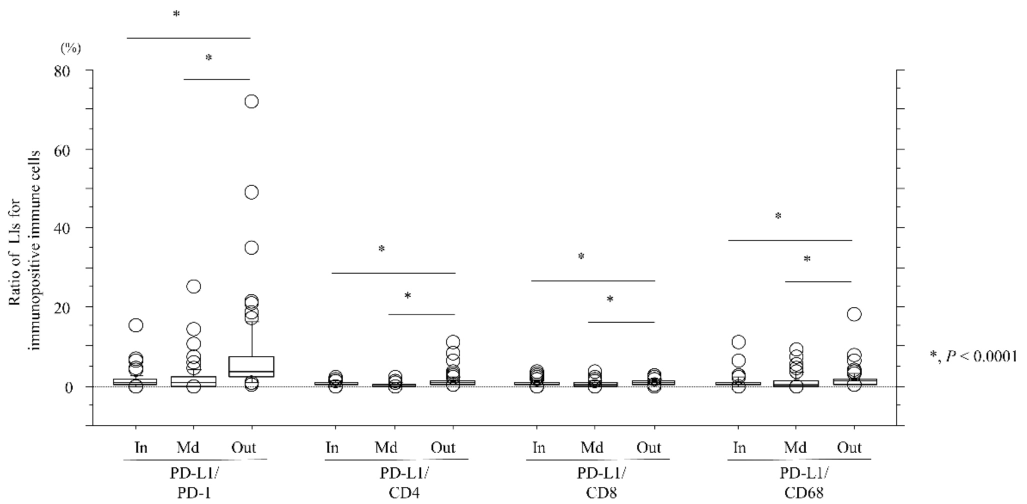

Figure S3. **Relationship between CD3, CD4, and CD8 in CRC without NCRT.** (A) Staining with hematoxylin and eosin (HE) or IHC for the indicated immune cell-related markers in CRC without NCRT. Original magnification, x100. (B) Correlations between the indicated markers in the outer stroma of CRC without NCRT.

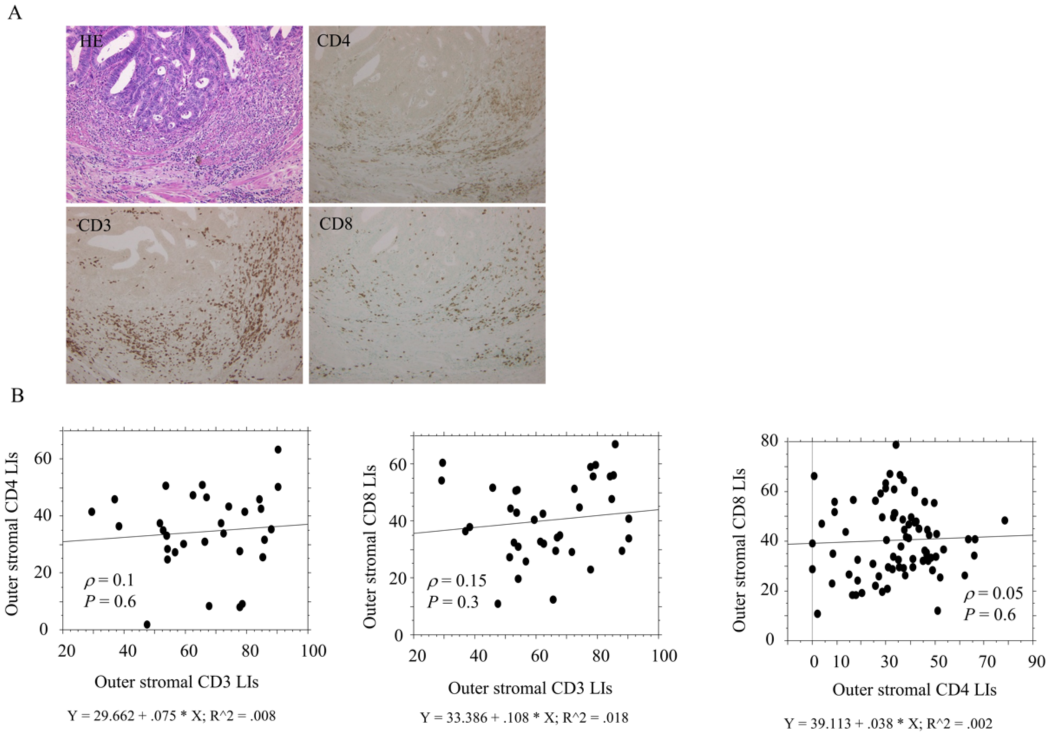

Figure S4. Ratios of PD-L1 LIs relative to PD-1, CD4, CD8, or CD68 LIs in the inner (In), middle (Md), and outer (Out) stroma in CRC without NCRT. The data shown are means ± SDs.

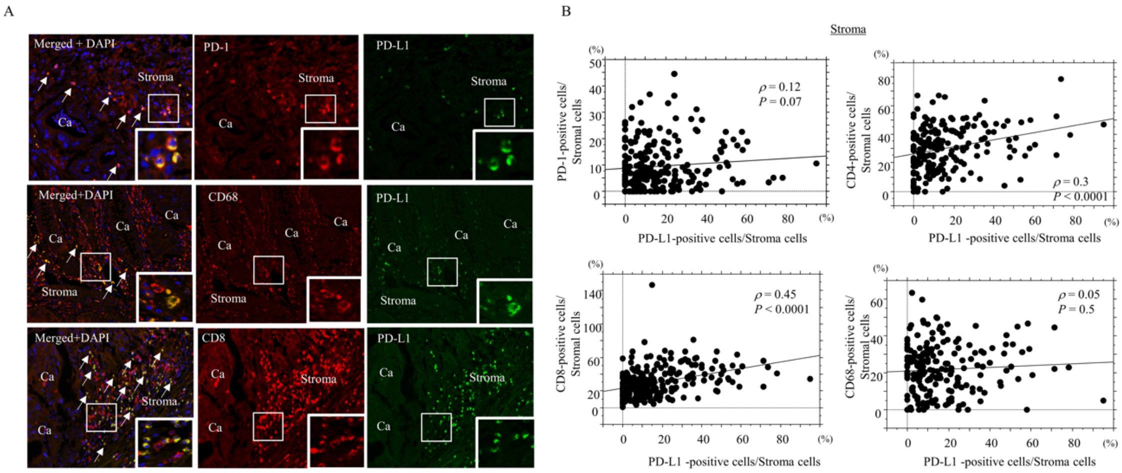

Figure S5. Coexpression of immune cell-related markers in infiltrating stromal immune cells in CRC without NCRT. (A) Immunofluorescence for PD-L1, PD-1, and CD68. Infiltrating immune cells coexpressing the indicated markers are present in the outer stroma (indicated by arrows). Closed boxes are magnified in the insets. Original magnification, x100 and x400 (insets). Ca, carcinomatous lesions. (B) Correlations between the indicated markers in the stroma of CRC samples.

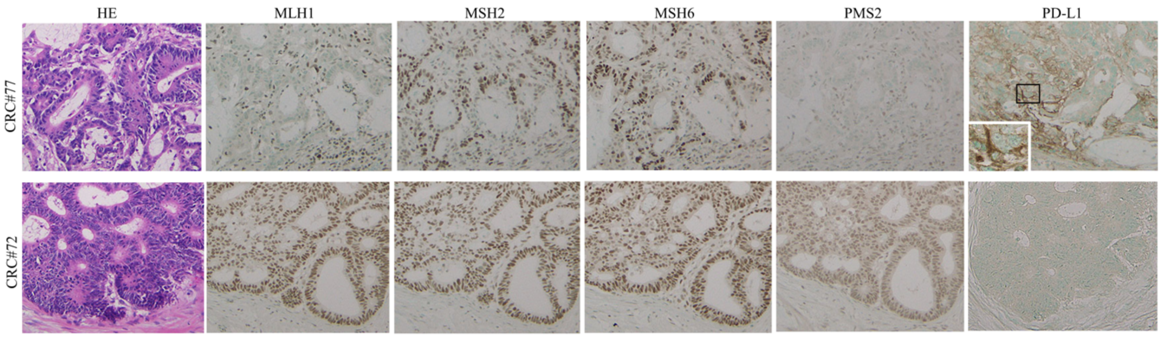

Figure S6. **Tumoral PD-L1 and MMR expression in CRC without NCRT.** Staining with hematoxylin and eosin (HE) and IHC for MMR and PD-L1 in CRC. Note that CRC case #77 (upper panels) is immunonegative for MLH1 and PMS2 in membranous PD-L1-positive tumor cells, which is in contrast to CRC case #72 which is PD-L1-negative and MMR-proficient. The closed box is magnified in the inset. Original magnification, x100 and x400 (inset).

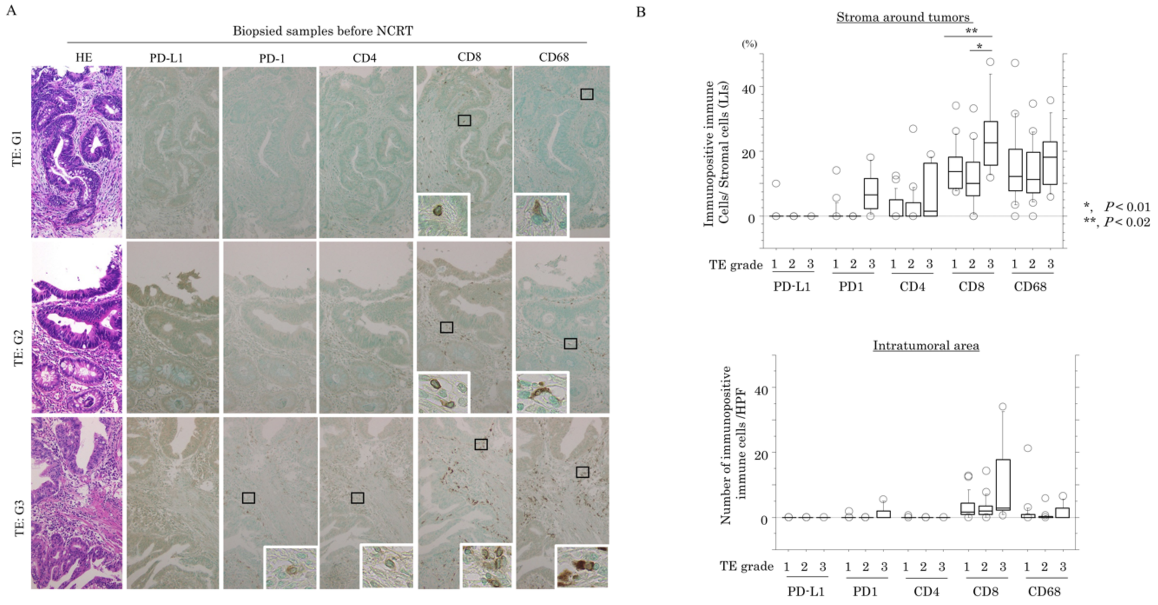

Figure S7. Expression of immune cell-related molecules in biopsied samples of LAd-RC before NCRT. (A) Staining with hematoxylin and eosin (HE) and IHC for the indicated immune cell-related markers in pretreatment biopsied LAd-RC samples that responded poorly to NCRT (TE: G1) (upper panels), responded moderately (TE: G2) (middle panels), or responded well (TE: G3) (lower panels) in the associated resected tumors after NCRT. Closed boxes are magnified in the insets. Original magnification, x100 and x400 (insets). (B) Relationships between labeling indices (LIs) of immunopositive immune cells for the indicated markers (upper), number of immune cells immunopositive for the indicated markers per high power field (HPF) in intratumoral lesions (lower) in pretreatment-biopsied LAd-RC samples, and TE grades in the associated resected tumor tissues after NCRT. The data shown are means ± SDs.

Table S1. Summary of antibodies used for immunohistochemistry

Antibody	Clone	Source	Dilution
PD-L1	28-8	Abcam (Cambridge, UK)	×700
PD1	NAT105	Abcam	×500
CD3	F7.2.38	DAKO (Copenhagen, Denmark)	x200
CD4	4B12	DAKO	×100
CD8	C8/144B	DAKO	×100
CD68	PG-M1	DAKO	×100
β-catenin	14/b-catenin	BD Bioscience (San Jose, CA, USA)	×500
MLH1	ES05	Novocastra (Newcastle, UK)	×100
MSH2	25D12	Novocastra	×50
MSH6	PU29	Novocastra	×100
PMS2	M0R4G	Novocastra	×100

Table S2. Association between carcinomatous and stromal PD-L1 and MMR status in CRC without NCRT

			PD-L1 expression						
			Carcinomatous lesion				Outer stromal lesion		
			high (≧5 %)	low (< 5%)			high LIs (≧30 %)	low LIs (< 30%)	
		n	n (%)	n (%)	*P*-value	n	n (%)	n (%)	*P*-value
MMR status	Proficient	90	5 (5.6)	85 (94.4)	0.0003	69	69 (80.2)	17 (19.8)	0.1
	Deficient	10	4 (40)	6 (60)		9	9 (100)	0	

n, number of cases

Table S3. Correlation between IHC marker expression and clinicopathological factors in the outer stroma in CRC without NCRT

		PD-1				CD4				CD8				CD68		
		high (≧8 %)	low (< 8 %)			high (≧34 %)	low (< 34 %)			high (≧41 %)	low (< 41 %)			high (≧27 %)	low (< 27 %)	
	n	n (%)	n (%)	*P*-value	n	n (%)	n (%)	*P*-value	n	n (%)	n (%)	*P*-value	n	n (%)	n (%)	*P*-value
Age (years)																
≧ 68	51	47(50.0)	4(4.3)	0.3	42	33(41.3)	9(11.3)	0.3	51	44(46.8)	7(7.4)	0.7	44	38(46.3)	6(7.3)	0.6
<68	43	37(39.4)	6(6.4)		38	33(41.3)	5(6.3)		43	36(38.3)	7(7.4)		38	31(37.8)	7(8.5)	
Gender																
Male	57	17 (18.1)	40 (42.6)	0.1	50	25 (31.3)	25 (31.3)	0.4	57	27 (28.7)	30 (21.9)	0.8	51	23 (28)	28 (34.1)	0.4
Female	37	16 (17)	21 (22.3)		30	18 (22.5)	12 (15)		37	17 (18)	20 (54)		31	17 (20.7)	14 (17.1)	
Location																
left side	39	17 (18.1)	22 (23.4)	0.1	35	17 (21.3)	18 (22.5)	0.4	40	21 (22.3)	19 (20.2)	0.3	35	15 (18.3)	20 (24.4)	0.3
right side	55	16 (17.0)	39 (41.5)		45	26 (32.5)	19 (23.8)		54	23 (24.5)	31 (33.0)		47	25 (30.5)	22 (26.8)	
Histology																
well/mod	79	28 (33.3)	51 (60.7)	0.8	66	31 (44.3)	35 (50)	0.03	79	38 (45.2)	41 (48.8)	0.2	66	30 (41.7)	36 (40)	0.8
muc/pap	5	2 (2.3)	3 (3.6)		4	4 (5.7)	0		5	1 (1.1)	4 (4.8)		6	3 (4.2)	3 (4.2)	
Clinical stage																
I	16	4 (4.3)	12 (12.7)	0.3	15	11 (13.8)	4 (5)	0.3	14	6 (6.4)	8 (8.5)	0.9	16	6 (7.3)	10 (12.2)	0.7
II	35	16 (17)	19 (20.2)		31	14 (17.5)	17 (21.3)		38	18 (193.1)	20 (21.3)		32	16 (19.5)	16 (19.5)	
IIIa	25	7 (7.4)	18 (19.1)		22	11 (13.8)	11 (13.8)		25	13 (13.8)	12 (12.8)		21	11 (13.4)	10 (12.2)	
IIIb	18	6 (6.4)	12 (12.8)		12	7 (8.8)	5 (6.3)		17	7 (7.4)	10 (10.6)		13	7 (8.5)	6 (7.3)	

LN meta																
Positive	43	13 (13.8)	30 (31.9)	0.3	34	18 (22.5)	16 (20)	0.9	42	20 (21.3)	22 (23.4)	0.8	34	18 (22)	16 (19.5)	0.5
Negative	51	20 (21.3)	31 (32.9)		46	25 (31.3)	21 (26.3)		52	24 (25.5)	28 (29.8)		48	22 (26.8)	26 (31.7)	
Ly infiltration																
Positive	68	23 (24.4)	45 (47.9)	0.6	59	31 (38.8)	28 (35)	0.7	68	34 (36.2)	34 (36.2)	0.3	61	29 (35.4)	32 (39)	0.7
Negative	26	10 (10.6)	16 (17)		21	12 (15)	9 (11.3)		26	10 (10.6)	16 (17)		21	11 (13.4)	10 (12.2)	
V infiltration																
Positive	79	26 (27.7)	53 (56.4)	0.3	66	36 (45)	30 (37)	0.7	79	37 (39.3)	42 (44.7)	0.9	67	35 (42.7)	32)40)	0.2
Negative	15	7 (7.4)	8 (8.5)		14	7 (8.8)	7 (8.8)		15	7 (7.4)	8 (8.5)		15		10 (12.2)	

well/mod, well/moderately differentiated; muc/pap, mucinous/papillary

n, number of cases; LN meta, lymph node metastasis; Ly, lymph vessel; V, venous vessel

Cut-off values for each category were defined as (mean - standard deviation) values

Clinical stage refers to the criteria of the Japanese Classification of Colorectal, Appendiceal and Anal Carcinoma

Table S4. Correlation between TE grade and BD score in LAd-RC after NCRT

		TE grade		
		1	2	*P*-value
	n	n (%)	n (%)	
BD score				
1	28	12 (24)	16 (32)	0.004
2	11	7 (14)	4 (8)	
3	11	11 (22)	0	

n, number of cases

Table S5. Univariate analysis for OS and PFS in LAd-RC after NCRT

Univariate analysis				
Variables	Cut-off	Log rank c2	*P*-value	Unfavorable factor
Overall survival				
PD-L1 (stroma)	1	3.9	0.06	
PD-1 (stroma)	1	0.038	0.84	
CD4 (stroma)	1	0.008	0.93	
CD8 (stroma)	3	0.004	0.95	
CD68 (stroma)	1	0.56	0.45	
MMR status	prof/def	0.0004	0.98	
Age	63/64	2.95	0.33	
Gender	Male/Female	0.45	0.5	
Pathological stage	<Ⅱ/>Ⅲ	6.05	0.018	>Ⅲ
LN metastasis	−/+	7.5	0.008	+
Ly involvement	−/+	0.47	0.49	
Venous involvement	−/+	1.67	0.2	
BD score	BD:1/BD:2,3	2.16	0.15	
Progression-free survival				
PD-L1 (stroma)	1	2.19	0.15	
PD-1 (stroma)	1	0.31	0.58	
CD4 (stroma)	1	0.001	0.98	
CD8 (stroma)	3	0.048	0.83	
CD68 (stroma)	1	0.67	0.4	
MMR status	prof/def	0.09	0.77	
Age	63/64	0.023	0.88	
Gender	Male/Female	1.24	0.27	
Pathological stage	<Ⅱ/>Ⅲ	10.9	0.001	>Ⅲ
LN metastasis	−/+	13.6	0.0005	+
Ly involvement	−/+	2.15	0.15	
Venous involvement	−/+	3.47	0.07	
BD score	BD:1/BD:2,3	1.73	0.19	

LN, lymph node; Ly, lymph vessel; prof/def, proficient/deficient; BD, budding

Cut-off values for each category were defined as (mean - standard deviation) values

Clinical stage refers to the criteria of the Japanese Classification of Colorectal, Appendiceal and Anal Carcinoma
